# Age, state, environment, and season dependence of senescence in body mass

**DOI:** 10.1002/ece3.3787

**Published:** 2018-01-18

**Authors:** Svenja B. Kroeger, Daniel T. Blumstein, Kenneth B. Armitage, Jane M. Reid, Julien G. A. Martin

**Affiliations:** ^1^ Institute of Biological and Environmental Sciences School of Biological Sciences Zoology Building University of Aberdeen Aberdeen UK; ^2^ Department of Ecology and Evolutionary Biology University of California Los Angeles CA USA; ^3^ The Rocky Mountain Biological Laboratory Crested Butte CO USA; ^4^ Ecology and Evolutionary Biology Department The University of Kansas Lawrence KS USA

**Keywords:** aging, altitude, late life, lifespan, natural population, reverse age

## Abstract

Senescence is a highly variable process that comprises both age‐dependent and state‐dependent components and can be greatly affected by environmental conditions. However, few studies have quantified the magnitude of age‐dependent and state‐dependent senescence in key life‐history traits across individuals inhabiting different spatially structured and seasonal environments. We used longitudinal data from wild female yellow‐bellied marmots (*Marmota flaviventer*), living in two adjacent environments that differ in elevation and associated phenology, to quantify how age and individual state, measured as “time to death,” affect body mass senescence in different environments. Further, we quantified how patterns of senescence differed between two biologically distinct seasons, spring, and late summer. Body mass senescence had an age‐dependent component, expressed as a decrease in mass in old age. Overall, estimated age‐dependent senescence was greater in females living in the more favorable lower elevation environment, than in the harsher higher elevation environment, and greater in late summer than in spring. Body mass senescence also had a state‐dependent component, captured by effects of time to death, but only in the more favorable lower elevation environment. In spring, body mass gradually decreased from 2 years before death, whereas in late summer, state‐dependent effects were expressed as a terminal decrease in body mass in the last year of life. Contrary to expectations, we found that senescence was more likely to be observed under more favorable environmental conditions, rather than under harsher conditions. By further demonstrating that senescence patterns differ among seasons, our results imply that within‐year temporal environmental variation must be considered alongside spatial environmental variation in order to characterize and understand the pattern and magnitude of senescence in wild populations.

## 1 INTRODUCTION

Senescence, defined as the gradual decrease in fitness traits with increasing age, results from deteriorating cellular and physiological function and thus the buildup of somatic damage (Kirkwood & Rose, 1991). Over the last few decades, the argument that wild animals do not senesce has been rebutted by a large number of empirical studies in various free‐living populations (reviewed by Nussey, Froy, Lemaître, Gaillard, & Austad, 2013). Classical evolutionary theories of senescence predict actuarial senescence (increase in mortality rate with increasing age) as a consequence of weakening natural selection with progressing chronological age (Medawar, 1952; Williams, 1957). This chronological age view has long dominated senescence studies in the field of evolutionary ecology. However, an emerging, complementary view is that individual state also plays an important role in defining senescence patterns (McNamara, Houston, Barta, Scheuerlein, & Fromhage, 2009; Monaghan, Charmantier, Nussey, & Ricklefs, 2008). Indeed, senescence is a highly heterogeneous within‐individual process that, for a given trait, can start at different ages (Gamelon et al., 2014; Péron, Gimenez, Charmantier, Gaillard, & Crochet, 2010), progress at different rates (Bouwhuis, Charmantier, Verhulst, & Sheldon, 2010a; Clutton‐Brock & Isvaran, 2007), and depend on environmental, early‐life, and natal conditions (Austad, 1993; Boonekamp, Salomons, Bouwhuis, Dijkstra, & Verhulst, 2014; Bouwhuis, Charmantier, Verhulst, & Sheldon, 2010b; Hammers, Richardson, Burke, & Komdeur, 2013; Hayward, Wilson, Pilkington, Pemberton, & Kruuk, 2009; Kim, Velando, Torres, & Drummond, 2011; Nussey, Kruuk, Morris, & Clutton‐Brock, 2007; Reed et al., 2008). At present, little is known about the relative importance of age‐dependent and state‐dependent components in shaping senescence trajectories, or the different spatiotemporal conditions that affect them.

The “age‐dependent component” of senescence quantifies trait variation as a function of chronological age, or time since birth (Nussey et al., 2011). From the perspective of a classical age‐dependent model, the age at onset and the rate of senescence are assumed to be similar across individuals. However, assumptions of this model preclude the possibility that senescence can occur in “young” individuals, below a certain “old” chronological age. The “state‐dependent component” (or “age independent,” Martin & Festa‐Bianchet, 2011; also “stage dependent’” in plants, Caswell & Salguero‐Gómez, 2013) of senescence, on the other hand, is based on the idea that the physiological state of an individual, and hence its “biological age,” may better explain late life decreases in performance (Kirkwood & Austad, 2000; McNamara et al., 2009). Thus, “chronologically young” individuals could be considered “biologically old” and also senesce. Differences in state arise as a result of different life‐history strategies and environmental conditions, generating differences in damage accumulation rates between individuals (McNamara & Houston, 1996; McNamara et al., 2009), and thus potential differences in age at onset and rates of senescence. For example, if reproduction leads to increased damage accumulation, fast reproducing individuals effectively bring forward their own death (McNamara et al., 2009). Increased damage accumulation implies that individuals have limited resources to allocate among competing systems of self‐maintenance and reproduction; this idea is also integral to the principle of allocation (Cody, 1966) and the disposable soma theory of senescence (Kirkwood, 1977; Kirkwood & Rose, 1991).

Studies using a state‐dependent approach to quantify late life variation in life‐history traits have frequently estimated “last year of life” effects, thereby quantifying terminal decreases or increases in phenotype (Bouwhuis, Sheldon, Verhulst, & Charmantier, 2009; Coulson & Fairweather, 2001; Froy, Phillips, Wood, Nussey, & Lewis, 2013; Hammers, Richardson, Burke, & Komdeur, 2012; Hayward et al., 2013; Nussey et al., 2011; Rattiste, 2004; Tafani et al., 2013; Weladji et al., 2006; Zhang, Vedder, Becker, & Bouwhuis, 2015). From a biological perspective, this approach may allow detection of terminal illness (Coulson & Fairweather, 2001), terminal investment (Williams, 1966) or terminal allocation (sensu Weladji et al., 2010; e.g., see Froy et al., 2013). It does not, however, allow detection of more gradual senescent decreases toward the end of life. Such effects can be quantified by estimating “time to death” effects and thus quantifying state‐dependent decreases several years before death (Martin & Festa‐Bianchet, 2011; Reed et al., 2008). While time to death is conceptually similar to other methods that allow senescence in chronologically young individuals, such as models that include age by lifespan interactions (Bouwhuis et al., 2009; Zhang et al., 2015), it is very different in its biological assumptions. An interaction between age and lifespan tests for varying senescence rates as a function of longevity, but because of the strong positive correlation between age and lifespan, this approach assumes a strong correlation between age at onset and rate of senescence (i.e., individuals with a later age at onset of senescence necessarily have faster rates). In a time to death model on the other hand, there is no covariation between age at onset and rate of senescence. The model assumes that age at onset differs across individuals, while individuals’ senescence rates are assumed to be similar and independent of longevity.

A decrease in performance with decreasing time to death would thus indicate onset of senescence independently of age per se, likely because individuals differ in state, which depends on among‐individual differences in life‐history strategies in their respective environment. However, as individuals’ state could also deteriorate and result in mortality without the underlying mechanism being senescence, time to death effects should be interpreted with care. Previously, time to death effects have been estimated when true age of individuals was unknown (Reed et al., 2008), or in addition to age effects, as they are not mutually exclusive (Hammers et al., 2012; Martin & Festa‐Bianchet, 2011). For example, in female bighorn sheep (*Ovis canadensis*), fecundity started decreasing 2 years before death, and at a faster rate in older individuals, indicating that individuals differed in both onset and rate of senescence (Martin & Festa‐Bianchet, 2011). However, relatively few ecological studies have estimated age and time to death effects simultaneously, likely because detailed longitudinal data for individuals of known age and lifespan are required but are challenging to obtain.

Senescence patterns could also be affected by environmental conditions experienced at different points in life (Austad, 1993; Bouwhuis et al., 2010a; Descamps, Boutin, Berteaux, McAdam, & Gaillard, 2008; Hämäläinen, Heistermann, & Kraus, 2015; Hayward et al., 2009; Millon, Petty, Little, & Lambin, 2011; Mumby et al., 2015; Nussey et al., 2007; Reed et al., 2008). For example, harsher environmental conditions have been linked to higher rates of age‐dependent actuarial and reproductive senescence in red deer (*Cervus elaphus*, Nussey et al., 2007), and higher rates of state‐dependent reproductive senescence in common guillemots (*Uria aalge*, Reed et al., 2008). Environmental conditions comprise factors like food abundance (Descamps et al., 2008), population density (Nussey et al., 2007), and seasonality (Hämäläinen et al., 2015), although the latter is rarely investigated. In general, few studies have explicitly tested for environmental effects on senescence trajectories, and even fewer have considered how different environmental conditions affect both onset and rate of senescence (but see, e.g., Beirne, Delahay, & Young, 2015; Hämäläinen et al., 2014).

In addition, most senescence studies in wild animals have focused on survival and reproduction, whereas considerably less attention has been devoted to physiological or morphological traits such as body mass (reviewed in Nussey et al., 2013), which may substantially affect survival and reproduction (Festa‐Bianchet, Gaillard, & Jorgenson, 1998; Gaillard, Festa‐Bianchet, Delorme, & Jorgenson, 2000). Recent studies in wild mammal populations have shown that body mass can exhibit varying degrees of senescence in reindeer (*Rangifer tarandus*; Weladji et al., 2010), bighorn sheep, roe deer (*Capreolus capreolus*) and Soay sheep (*Ovis aries*, Nussey et al., 2011; Hayward et al., 2015; Douhard, Gaillard, Pellerin, Jacob, & Lemaître, 2017), Alpine marmots (*Marmota marmota*; Tafani et al., 2013), mouse lemurs (*Microcebus murinus*; Hämäläinen et al., 2014), and European badgers (*Meles meles*; Beirne et al., 2015). Some of these studies explicitly tested for terminal effects (Beirne et al., 2015; Douhard et al., 2017; Hämäläinen et al., 2014; Nussey et al., 2011; Tafani et al., 2013), and two studies quantified time to death effects, thereby testing for gradual state‐dependent senescence, but only found evidence for more sudden terminal decreases over the last year of life (Hayward et al., 2015; Nussey et al., 2011). What was not considered by recent studies is that, because body mass varies with environmental conditions, which in turn vary with seasonal timing, observation of body mass decreases may also depend on the time of year at which body mass is measured. This is especially relevant in hibernating species in which body mass is a particularly critical trait. Hibernators undergo strong seasonality and can exhibit striking differences between pre‐ and posthibernation body masses. Consequently, body mass decreases may be more likely to be observed if body mass was measured preceding the food‐scarce season, as individuals that lose a lot of mass may die during hibernation and thus be absent from subsequently collected samples (as suggested in bighorn ewes, Bérubé, Festa‐Bianchet, & Jorgenson, 1999).

Here, we used long‐term individual‐based data from a population of yellow‐bellied marmots (*Marmota flaviventer*) around the Rocky Mountain Biological Laboratory, Colorado, to quantify female age‐dependent and state‐dependent body mass senescence, and to test how different environmental conditions and seasonal timing affect senescence patterns. Marmots in this population hibernate for about 7 months per year and exhibit up to forty percent differences between spring (postemergence) and late summer (prehibernation) body masses (Armitage, 2014). Unsuccessful hibernation is a major cause of death in the marmots (Schwartz, Armitage, & Vuren, 1998), and lower mass at entry into hibernation is negatively associated with overwinter survival (Armitage, 1994; Armitage, Downhower, & Svendsen, 1976; Lenihan & Vuren, 1996). As individuals were monitored from birth to death and repeatedly weighed within and across different years, we could test for age and time to death effects on body mass in both spring and late summer. Further, marmots were monitored in two areas at different elevations that differ in phenology and ecology (Blumstein, Im, Nicodemus, & Zugmeyer, 2004; Kilgore & Armitage, 1978), allowing us to test for environmental effects on senescence trajectories.

We tested three sets of nonmutually exclusive hypotheses. First, following classical senescence theory, we hypothesized that body mass senescence has an age‐dependent component, expressed as a decrease in mass with increasing age, and implying senescence at old chronological ages. Higher elevation environments commonly pose harsher or more constraining conditions than lower elevation environments, due to colder temperatures leading to later food availability, shorter reproductive seasons, and higher thermoregulatory costs (Bears, Martin, & White, 2009). We therefore predicted higher rates of age‐dependent senescence in the harsher higher elevation environment.

Second, if damage accumulation rates differ among individuals as a result of different life histories (McNamara et al., 2009) and in line with disposable soma theory, senescence should be at least partly independent of age. We thus hypothesized that body mass senescence also has a state‐dependent component, expressed as a gradual decrease with decreasing individual time to death, and/or as a terminal decrease in the last year of life. As disposable soma effects are expected to be stronger under harsher environmental conditions, where reproduction is increasingly favored over somatic maintenance (Kirkwood, 1977), we predicted higher rates and earlier onset of state‐dependent senescence in the harsher higher elevation environment.

Third, as body mass varies markedly among different seasons, patterns of body mass senescence may differ depending on when body mass is measured. Because light individuals are more likely to die during hibernation than during the active period (Armitage et al., 1976; Armitage, 1994, 2014, pp. 97–104), and therefore may be absent from spring samples, we hypothesized that age‐dependent and state‐dependent effects on body mass are greater in late summer. Specifically, we predicted terminal effects to be absent in spring, as individuals with substantial mass loss in the last year of life are particularly unlikely to survive hibernation.

## 2 MATERIALS AND METHODS

### 2.1 Study system and environment

Since 1962, a population of yellow‐bellied marmots was studied along a 5‐km stretch of the Upper East River Valley, Colorado, (38°57′N, 106°59′W; 2,900 m elevation; Armitage, 2014). Colony sites in the study area are grouped into “up‐valley” (higher elevation) and “down‐valley” (lower elevation), reflecting an elevational difference of 165 m. Snowmelt date and onset of vegetation growth are delayed up‐valley (Blumstein et al., 2004; Armitage, 2014; pp. 119–129), where marmots emerge from hibernation two weeks later than down‐valley on average (emergence between mid‐April and mid‐May; Blumstein, 2009; Monclús, Pang, & Blumstein, 2014). Females have a maximum of one litter per year, between mid‐May and mid‐June, and pups are weaned and fully independent after a lactation period of 25–35 days (Armitage, 2014). Individuals rarely move between the two valley areas, and all females included in this study experienced local environmental conditions of either up‐valley or down‐valley throughout their lives.

### 2.2 Body mass

Every year during the active period, between mid‐May and mid‐September, marmots were trapped at known burrow locations using single‐door live traps (81 × 25 × 30 cm, Tomahawk Live Trap Co., Wisconsin, USA) baited with horse feed or oats. Captured individuals were weighed using a handling bag and digital suspended scales (accuracy <50 g), and sex was recorded. To allow subsequent identification within and across years, each individual was tagged with two uniquely numbered metal ear tags at first capture (1005–3 Monel self‐piercing fish tags). Individuals were also dorsally fur marked with nontoxic black dye to allow remote identification.

During 1975–2013, to attempt to capture each adult (i.e., age ≥3 years) at least once every two weeks, weekly trapping sessions alternated between up‐valley and down‐valley colonies. To allow comparison of spring and late summer body masses among years, measures taken in each year (between mid‐May and mid‐September, over a range of dates) were standardized to 1 June and 15 August (Martin & Pelletier, 2011; Ozgul et al., 2010). June mass reflects the trade‐off between energy used during hibernation and that available for reproduction (Armitage, 2014). As masses of unborn litters are very small compared to normal diurnal variation in female body mass (Frase & Hoffmann, 1980), pregnancy was not detectable in females’ June body masses and hence did not affect estimates of female mass trajectories. August mass reflects fat mass gain during the active period. It predicts overwinter survival and affects reproductive success the following year by determining June mass to some degree (Armitage, 2014).

Best linear unbiased predictors (BLUPSs) from linear mixed models were used to obtain the standardized body masses for each individual in each season in each year (following Ozgul et al., 2010; Maldonado‐Chaparro, Martin, Armitage, Oli, & Blumstein, 2015; Appendix S1). Although analyzing BLUPs as individual standardized metrics can cause biases (Hadfield, Wilson, Garant, Sheldon, & Kruuk, 2010), they provide more accurate individual estimates than least squares regression models, especially when individuals have few observations (Martin & Pelletier, 2011). To minimize extrapolation of body mass estimates from measurements that were far from the 1 June and 15 August standardization points, June and August estimates from females that were first weighed in August or not weighed after June, respectively, were excluded. Individuals that were only weighed once more than 4 weeks away from both standardization points were also excluded. June and August mass are inherently correlated because adults have a constant skeletal size after age 3. However, due to individual variation in summer fat accumulation, correlations were less than one, meaning that seasonal masses can be considered different traits (Pearson's correlation: *r* = .75; *N* = 472 individual‐year observations). As the current aim was to contrast body mass senescence patterns between seasons, analyses focused on June and August mass as two separate traits rather than the difference between the two. June and August mass are hereafter referred to as spring and late summer mass, respectively.

As marmots reach their full adult body size by age three, subsequent body mass variation, particularly within years, primarily reflects variation in fat mass (Armitage, 2014; pp. 97–104). Variation in body mass across years, however, may also reflect the buildup or breakdown of other tissues that were not measured in this study. Consequently, observed senescent decreases in body mass may underlie a combination of mechanisms; not only could individuals accumulate fewer fat reserves over the summer (Yearsley et al., 2005), but damage may accumulate in a range of somatic tissues (Kirkwood & Austad, 2000; Monaghan, Metcalfe, & Torres, 2009), including muscle tissue for example (Hindle, Lawler, Campbell, & Horning, 2009).

Two‐year olds are also sexually mature and hence adults, but as they have not reached their full skeletal size, they were excluded from analyses to avoid confounding effects of growth on body mass variation. Also, based on hind‐foot length, skeletal size did not differ between females living up‐valley versus down‐valley, thus any between‐valley differences in body mass are unlikely to be due to differences in skeletal size.

### 2.3 Age, lifespan, and time to death

Only females of known age (first captured as pups) and lifespan were included in the data set. Lifespan was calculated as each female's age at last observation, so for example, an individual that was last seen in 2001 at age 5, but is not observed in 2002 or thereafter, has a lifespan of 5 years. As the recapture probability of adult marmots has been estimated to exceed 98% using multistate mark–recapture analyses (Ozgul, Armitage, Blumstein, & Oli, 2006; Ozgul, Oli, Olson, Blumstein, & Armitage, 2007), and untrapped marked females were identified as alive during weekly colony monitoring, these estimates are accurate. To minimize potential biases stemming from selective disappearance, all individuals from nonextinct cohorts were excluded, except that 55 individuals from five cohorts were retained, for which a maximum of two individuals were still alive (seven individuals in total). Time to death was calculated by subtracting age from lifespan in every year of a female's life.

### 2.4 Statistical models

For each season, a separate mixed model was fitted to quantify environment‐specific age‐dependent and state‐dependent senescence in body mass. To quantify variation in body mass with chronological age, both season‐specific models included linear and quadratic effects of age. To further examine whether significant quadratic age effects described a decrease in body mass in old age, rather than an increase at young age followed by a plateau, a linear regression was fitted to the subset of individuals that were weighed beyond the age at which body mass reached its predicted maximum (hereafter “old females”). This cutoff point does not necessarily reflect the exact age at onset of age‐dependent senescence, but allows a straightforward test for a decrease in mass at old chronological ages.

To test for changes in body mass in the years before death, both season‐specific models also included a time to death (TTD) effect, modeled as a four‐level factor (0, 1, 2, and 3 years or more before death) where the level TTD0 corresponds to the last year of life.

To control for differences in body mass between environments, both season‐specific models included a two‐level valley factor (i.e., up‐valley versus down‐valley). To test for effects of environmental conditions on age‐ and state‐dependent variation in body mass, both models also included two‐way interactions between valley and linear and quadratic effects of age, and between valley and TTD. To test whether state‐dependent effects are only expressed at certain ages, models additionally included two‐way interactions between TTD and linear and quadratic effects of age.

To statistically account for selective disappearance (Vaupel, Manton, & Stallard, 1979; Vaupel & Yashin, 1985) in either or both valley environments, both season‐specific models additionally included linear effects of lifespan (Van de Pol & Verhulst, 2006), and a two‐way interaction between valley and lifespan. A positive relationship between body mass and lifespan would indicate higher mortality of lighter individuals, causing increasing body mass with age at the population level. The high capture probability in this population means that there is little opportunity for capture success to vary with body mass; therefore, patterns of variation in body mass are not driven by weight‐selective trapping.

As reproduction in spring affects body mass because lactating females allocate resources to their pups and start accumulating fat reserves later than nonreproducing females (Armitage, 2014, pp. 98–100), we also accounted for current year reproduction by including a two‐level factor (“litter”: yes or no) for reproductive success. A female was considered successful if she weaned at least one pup in a given year.

Full models are presented, including nonsignificant fixed effects (Whittingham, Stephens, Bradbury, & Freckleton, 2006); however, non‐significant interactions (*p* > .05) were backwards eliminated using ANOVAs with Satterthwaite approximation for the number of degrees of freedom, to ensure that they did not bias other estimates (Engqvist, 2005). Models that were further reduced to only significant effects provided quantitatively similar estimates for retained terms. To facilitate interpretability of coefficients, age and lifespan were centered with a mean of 0.

Random individual identity and year effects were fitted in all models. All models were fitted in R 3.1.1 (R Development Core team, 2014) using the lme4 package (Bates et al., 2014). We present estimated effects with 95% confidence intervals, which were determined using profile likelihoods (Bates et al., 2014).

The robustness of age‐dependent effects was confirmed via additional analyses with reduced data sets that excluded observations of individuals aged either 10, 11, or 12 and older, providing qualitatively similar results. To test for between‐season differences in body mass variation with age, TTD, and lifespan directly, and thus verify apparent between‐season differences in these effects, we fitted further models to data sets where we combined the spring and late summer data and split them by up‐valley versus down‐valley. One model per valley was then fitted with fixed season effects, and interactions between season with age, TTD, and lifespan. Backwards eliminations of nonsignificant interactions and further examination of significant quadratic age effects were carried out as above.

Modeling lifespan effects was necessary to account for selective disappearance (Van de Pol & Verhulst, 2006). However, lifespan and age are inevitably correlated because old ages can only be reached by long‐lived individuals. To verify that collinearity between age and lifespan did not affect the results, models were refitted with data split into three discrete lifespan categories (Reid, Bignal, Bignal, McCracken, & Monaghan, 2003), defined as short‐lived (4–6 years), medium‐lived (7–9 years), and long‐lived (10–14 years). Females with lifespans of 3 years were excluded from the short‐lived category because they only had 1 year of data and therefore provided no information on body mass variation with age or TTD.

## 3 RESULTS

The data sets for spring and late summer included 590 and 475 body mass estimates from 203 and 171 individual females, respectively. Across both data sets, there were a total of 205 different females (78 living down‐valley and 127 living up‐valley), of which twenty‐five were weighed only once as fully grown adults (i.e., aged ≥3 years). The remaining 180 females were weighed over a range of years, with a mean of 3.8 weighings within a year. The mean age across all female‐years was 5.1 years (median = 4, interquartile range: IQR = 3–6), with lifespans ranging between 3 and 14 years (down‐valley: mean = 6.3, median = 6, IQR = 4‐8; up‐valley: mean = 5.3, median = 5, IQR = 3‐6). On average, females living up‐valley were 300–400 g lighter than females living down‐valley and mean body mass was 900 g greater in late summer than in spring. Full distributions of spring and late summer body masses in each valley are shown in Figure S1.

### 3.1 Age‐dependent variation

**Figure 1:**
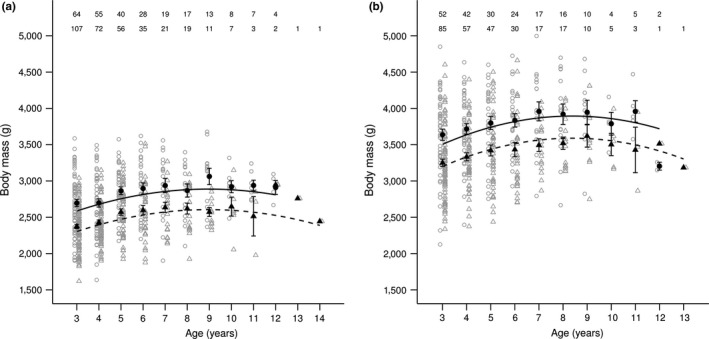
Relationships between body mass and age (in years) in (a) spring and (b) late summer, in female yellow‐bellied marmots living (solid lines, circles) down‐valley and (dashed lines, triangles) up‐valley. Black lines are predictions from the models in Table 1. Filled circles and triangles (±*SE*) show mean body masses per age and open circles and triangles show the raw data, down‐valley, and up‐valley, respectively. Sample sizes are shown for each age class, for females living (top row) down‐valley and (bottom row) up‐valley

**Figure 2:**
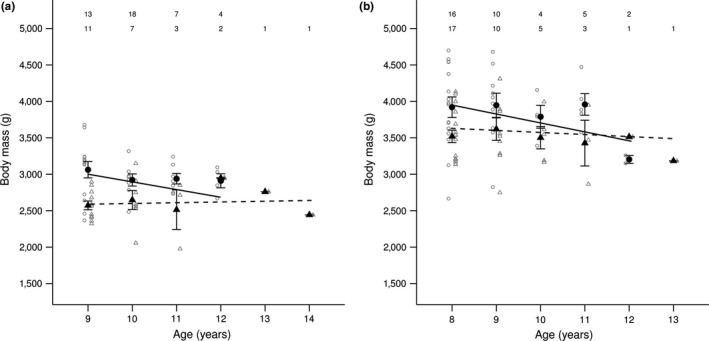
Relationships between body mass and age (in years) in (a) spring and (b) late summer, in old female yellow‐bellied marmots living (solid lines, circles) down‐valley and (dashed lines, triangles) up‐valley. Lines are predictions from the linear models in Table S1. Filled circles and triangles (±*SE*) show mean body masses, and open circles and triangles show the raw data, down‐valley, and up‐valley, respectively. Sample sizes in each season are shown for each age class, for old females living (top row) down‐valley and (bottom row) up‐valley

In both spring and late summer, there were positive linear and negative quadratic effects of age on body mass (Figure 1; Table 1). Linear regressions fitted across “old females,” weighed at and beyond age 9 and 8, in spring and late summer, respectively (predicted ages at maximum body mass: spring = 9.8 years; late summer = 8.4 years), showed that negative quadratic effects did describe body mass decreases in old age. Spring body mass decreased only down‐valley (Figure 2a; Table S1), whereas late summer body mass decreased both up‐valley and down‐valley (Figure 2b; Table S1). Thus, there was evidence of age‐dependent senescence in spring body mass down‐valley but not up‐valley, and in late summer body mass in both valley areas.

**Table 1:** Linear mixed‐effects models quantifying effects of age, time to death (TTD), valley, lifespan, and reproduction (litter) on spring and late summer body mass in female yellow‐bellied marmots

Fixed effect	*N* = 590; 203 individuals	*N* = 475; 171 individuals
	Spring	Late summer
	Estimate (95% CI)	Estimate (95% CI)
Intercept	2642.49 (2537.7/2746.7)*	3788.63 (3646.7/3929.9)*
Age	152.58 (120.3/184.9)*	225.22 (159.3/291.1)*
Age^2^	−8.52 (−10.7/−6.3)*	−13.43 (−18.2/−8.6)*
TTD[1]	40.01 (−19.7/99.6)	88.05 (4.7/171.3)*
TTD[2]	134.96 (65.8/204.1)*	4.99 (−94.3/104.3)
TTD[3+]	150.64 (69.0/232.2)*	155.87 (21.5/290.2)*
Valley[up]	−161.99 (−239.0/−84.1)*	−317.74 (−431.3/−203.7)*
Lifespan	−4.10 (−22.3/14.1)	−14.22 (−46.4/17.8)
Litter[yes]	−37.99 (−68.7/−7.2)*	−304.28 (−364.4/−244.0)*
TTD[1] × Valley[up]	−22.88 (−97.2/51.5)	
TTD[2] × Valley[up]	−152.48 (−234.5/−70.5)*	
TTD[3+] × Valley[up]	−133.04 (−208.8/−57.3)*	

Eliminated interaction terms are shown in Table S4. The reference levels for TTD, valley, and litter are [0], [down], and [no], respectively. TTD[0] corresponds to the last year of life, and TTD[3+] denotes 3 years or more before death. Random effects variances of “female identity” and “year observed” are 48,311 and 58,444 in spring, and 104,663 and 39,255 in late summer. Terms for which 95% confidence intervals (CI) did not overlap zero are denoted with an asterisk (*).

Valley‐specific models, which combined data from both seasons and tested for between‐season differences in body mass variation with age, TTD, and lifespan directly, confirmed the results obtained from the season‐specific models (Tables S2, S3).

### 3.2 State‐dependent variation

**Figure 3:**
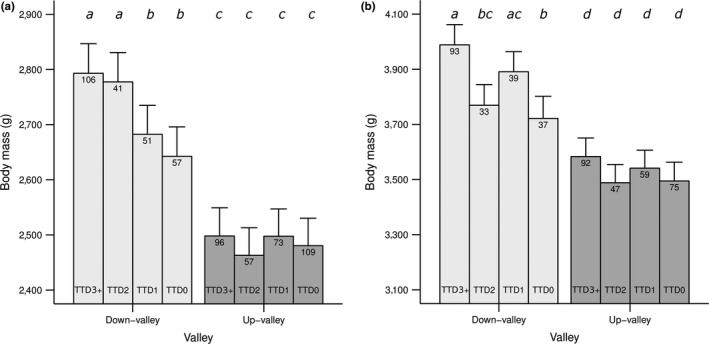
Predicted values of body mass with time to death (TTD) in (a) spring and (b) late summer, in female yellow‐bellied marmots living (light‐gray) down‐valley and (dark‐gray) up‐valley. Bars (±*SE*) are predictions from the models in Table 1. The levels in TTD are 3 years or more (TTD3+), 2 years (TTD2), 1 year (TTD1), and 0 years (TTD0) before death, where TTD0 equates to the last year of life. Different lowercase letters in each season indicate significant body mass differences among years to death, and between valleys. Sample sizes in each season are shown for each time to death class in each valley

In spring, there was a TTD by valley interaction (Table 1). This interaction showed that female body mass decreased from 2 years before death (TTD2) down‐valley but not up‐valley (Figure 3a; Table 1). Body mass was not significantly different between the year before death (TTD1) and the last year of life (TTD0) in either part of the valley. Thus, there was no evidence for an additional terminal decrease in body mass close to death. Further, there was no TTD by age interaction and thus no evidence that time to death effects on body mass vary with age (Table S4).

In late summer, there was a TTD effect on body mass in both valley areas (Table 1). Females were lighter in their last year of life (TTD0) compared to the previous year (TTD1; Figure 3b; Table 1). Thus, there was a terminal decrease in body mass close to death, but no gradual decrease over several years preceding death. Again, there was no TTD by age interaction (Table S4).

The valley‐specific model, which combined data from both seasons and tested for between‐season differences in body mass variation with TTD directly, confirmed that down‐valley, the TTD effect was indeed significant and differed between seasons (Table S2; ANOVA: “TTD*season” interaction, *F*_3,348.1_ = 4.5, *p* < .01). However, the valley‐specific model for up‐valley showed that TTD effects were only significant in spring and not in late summer (Table S2). Thus, in both seasons, we found evidence for state‐dependent body mass decreases down‐valley but not up‐valley.

No significant effects of female lifespan on body mass were found in either season or valley (Table 1), suggesting that there was no selective disappearance. This result was supported by models fitted to subsets of data comprising three discrete lifespan categories, which showed qualitatively similar results for effects of age and TTD on body mass across short‐, medium‐, and long‐lived lifespan categories, and compared to models fitted to the full data set (Tables S5, S6). Lastly, as expected, there was an effect of reproduction on body mass in both seasons, showing that females which had successfully weaned a litter in a given year had lower body masses.

## 4 DISCUSSION

Few studies have simultaneously quantified age‐dependent and state‐dependent senescence in key life‐history traits such as body mass across different spatially structured and seasonal environments. We show that effects of age and time to death on body mass co‐occur, and that their expression differs among seasons and exhibits strong environment dependence. Overall, and opposite to expectation, age‐dependent senescence and state‐dependent senescence (i.e., time to death effects) were more likely to be observed at lower elevation than higher elevation, and thus under more favorable rather than harsher conditions. These findings imply that inference of senescence patterns should not be drawn solely from phenotypes measured in single locations and seasons, as this may lead to very localized and time‐dependent conclusions regarding the extent of life‐history variation and associated evolutionary dynamics.

### 4.1 Age‐dependent variation

Our results show that body mass senescence had an age‐dependent component, implying senescence at old chronological ages. In spring, a decrease in body mass of old females was exhibited at lower elevation (down‐valley). The age‐dependent decrease is in line with predictions from classical senescence theories (Medawar, 1952; Williams, 1957) and concurs with other studies that found body mass senescence in mammals (Beirne et al., 2015; Hämäläinen et al., 2014; Nussey et al., 2011; Tafani et al., 2013; Weladji et al., 2010). Indeed, age‐dependent body mass senescence has previously been found in a closely related species, the Alpine marmot (Tafani et al., 2013). However, Tafani et al. (2013) only observed senescence in males, not in females. Differences in senescence patterns between Alpine and yellow‐bellied marmot females likely reflect species‐specific physiological and environmental conditions (see Section 2.2 in “state‐dependent variation”).

We did not observe the age‐dependent spring mass decrease in old females living in the higher elevation (up‐valley) environment with the shorter growing season, which was predicted to exhibit higher rates of senescence than the lower elevation environment. The differences in age‐dependent senescence between the two elevations probably reflect different environment‐specific optimization trade‐offs (Krebs & Davies, 1997; Stearns, 1992). If the higher elevation environment is harsher, females might be subject to environmentally induced physiological constraints (Curio, 1983), and thus exhibit little senescence because they have little mass to lose (Pelletier, Réale, Garant, Coltman, & Festa‐Bianchet, 2007). For example, heavier female gray seals (*Halichoerus grypus*) weaned larger, fatter pups, and exhibited greater mass loss during the lactation period compared to lighter females (Iverson, Bowen, Boness, & Oftedal, 1993). Indeed, our study showed that female marmots living at higher elevation had lower life‐long mean body masses than females living at lower elevation. The effect of a constraint on body mass should be further exacerbated in spring following the hibernation period, as females that lost relatively more body mass over the winter probably died and are thus absent from spring samples. Similarly, senescent decreases in body mass were more pronounced in September than in June in bighorn sheep (Bérubé et al., 1999). In addition, if female marmots are senescing, they may also accumulate less fat during the active period. Age‐dependent senescence would then more likely be observed in late summer, when females reach their peak annual mass. Indeed, we found that in late summer, old females exhibited significant age‐dependent decreases in body mass in both environments. However, the estimated effect of age at higher elevation was small (see Figure 2b; Table S1), indicating a lower rate of senescence in the harsher environment compared to the more favorable lower elevation environment.

### 4.2 State‐dependent variation

We also found that body mass senescence had a state‐dependent component, captured by “time to death” effects. This suggests that the age at which decreases in body mass commence differs among individuals. In spring, body mass of females living in the lower elevation environment decreased over the last 2–3 years prior to death, independently of age. This indicates that females experience increased damage accumulation toward the end of life, possibly due to increased reproductive effort and/or increased maintenance and reproductive costs (Kirkwood, 1977; McNamara et al., 2009). These results match predictions of the damage accumulation model by McNamara et al. (2009), which also fits with the disposable soma theory (Kirkwood, 1977). To date, no previous studies have found gradual state‐dependent senescence in body mass, but other studies provide evidence for such effects in reproductive traits (common guillemots, Reed et al., 2008; mute swans, *Cygnus olor*, McCleery, Perrins, Sheldon, & Charmantier, 2008; bighorn sheep, Martin & Festa‐Bianchet, 2011; European badgers, Dugdale, Pope, Newman, Macdonald, & Burke, 2011). However, in late summer, state‐dependent effects were expressed as a terminal decrease in mass over the last year of life, again only in females living at lower elevation. This result is consistent with most other recent studies on body mass senescence, which also reported terminal effects (Beirne et al., 2015; Douhard et al., 2017; Hämäläinen et al., 2014; Hammers et al., 2012; Nussey et al., 2011; Tafani et al., 2013). While a variety of factors could potentially lead to terminal decreases, it is commonly suggested that they indicate terminal illness, causing a sudden collapse in condition prior to death (Coulson & Fairweather, 2001). Alternatively, they may reflect reproductive decisions such as terminal investment (Clutton‐Brock, 1984; Williams, 1966) where greater reproductive investment at the beginning of the active period may cause a subsequent decrease in body mass and increased mortality risk (Pianka & Parker, 1975; but see Festa‐Bianchet et al., 1998). In Alpine marmots, body mass also decreased terminally in the year preceding death, but only in males (Tafani et al., 2013). As Alpine and yellow‐bellied marmots exhibit differences in their ecology (Tafani, Cohas, Bonenfant, Gaillard, & Allainé, 2012), the absence of terminal effects in female Alpine marmots is not necessarily surprising. Alpine marmot females may, however, experience broadly similar environmental conditions to yellow‐bellied marmot females living in the harsher higher elevation environment, rather than the more favorable lower elevation environment, and thus might also be closer to the required body mass threshold for surviving hibernation. In fact, Tafani et al. (2012) suggested that Alpine marmots are less efficient hibernators than yellow‐bellied marmots and thus they could drop below the required body mass threshold even faster. Tafani et al. (2013) did not test for environmental effects, but acknowledged the potential importance of environmental conditions in determining patterns of senescence.

Disposable soma effects are expected to be stronger in harsher environments due to higher extrinsic mortality (Kirkwood, 1977), and accordingly females in the higher elevation environment were predicted to exhibit higher rates and earlier onset of state‐dependent senescence. However, contrary to predictions, and similar to age‐dependent effects, we primarily found state‐dependent effects in females living in the lower elevation environment. A few previous studies have also found greater intensity and earlier onset of age‐dependent senescence under more favorable versus less favorable conditions (Douhard et al., 2017; Hämäläinen et al., 2014). In those cases, selective disappearance appeared to be a critical factor. However, we did not find evidence for selective disappearance in the marmots. Again, if body mass of females at higher elevation is constrained, decreases in mass are less likely to be observed because females quickly drop below the threshold of required body mass to survive hibernation. Our results in females at higher elevation match findings from other studies that investigated life‐history differences among elevations. For example, dark‐eyed juncos (*Junco hyemalis*) at higher elevations followed a high‐survival strategy compared to a high‐reproduction strategy in individuals at lower elevations (Bears et al., 2009). Thus, the most parsimonious explanation for the absence of state‐dependent body mass decreases in females living at higher elevation may be that they are less able to reproduce toward the end of their lifespan, possibly due to increased maintenance and/or reproductive costs and reproductive senescence.

Interestingly, the estimated state‐dependent effects on female yellow‐bellied marmot body mass at lower elevation were expressed as a gradual decrease in spring versus a terminal decrease in late summer. This result is consistent with the prediction that terminal effects are more likely to be observed in late summer, because individuals with high mass loss in their last year of life likely died during hibernation and consequently are absent from spring samples. Within a given year, mass in late summer is strongly affected by reproductive decisions and fat accumulation over the summer. While the gradual state‐dependent decrease in spring mass could reflect deterioration of various somatic tissues other than fat (e.g., muscle tissue, Hindle et al., 2009), we might not have detected this effect in late summer mass because of increased fat accumulation over the active period in late life. It is possible that due to reproductive senescence, female marmots fail at reproduction in spring and consequently have more time to accumulate fat. Alternatively, if females adopt a conservative strategy to maximize reproductive output (Stearns, 1992), they may favor fat storage and survival by skipping reproduction in some years (Armitage, 2014). These hypotheses remain to be explicitly tested.

Overall, this study shows that environmental conditions can have profound impacts on senescence trajectories and can generate different senescence patterns among individuals within the same population. Studies on the mechanisms underlying the observed patterns would now be interesting to elucidate the reasons for the absence of senescence effects at higher elevation.

## DATA ACCESSIBILITY

Data for this study have been archived in the Dryad Digital Repository https://doi.org/10.5061/dryad.sv26t.

## CONFLICT OF INTEREST

None declared.

## AUTHORS’ CONTRIBUTIONS

The study was designed by S.B.K., J.G.A.M., and J.M.R.; S.B.K., D.T.B., K.B.A., and J.G.A.M. collected the data. S.B.K. analyzed the data and wrote the manuscript, with input from all authors.
