# Supplementary material for: Age, state, environment, and season dependence of senescence in body mass
Source: Ecol Evol. 2018 Jan 18;8(4):2050–61. doi: 10.1002/ece3.3787 (PMC5817150; doi:10.1002/ece3.3787)
Supplement: Supplementary file 1 [file ECE3-8-2050-s001.docx]

**ONLINE SUPPLEMENT:**

**Appendix S1. Methods for body mass estimation models**.

We used linear random regression models to standardise body masses for each individual in each season in each year. The models included linear age effects, and linear and quadratic day of year effects (Ozgul *et al*. 2010; Martin & Pelletier 2011), and random individual identity, year and site effects. In addition, random deviations for both linear and quadratic effects of day of the year were fitted for each random effect. Since the goal of modelling was prediction, we did not attempt to determine whether specific variance components were significantly different from zero. Negligible sources of variation are estimated to be near zero and thus contribute little to predicted values. The fitted models were used to predict 1^st^ June (i.e. ‘spring’) and 15^th^ August (i.e. ‘late summer’) body masses for each individual in each year, conditional on the predicted random effects given by the best linear unbiased predictors (BLUPs).

Literature Cited

Martin, J.G.A., & Pelletier, F. (2011). Measuring growth patterns in the field: effects of sampling regime and methods on standardized estimates. *Canadian Journal of Zoology*, **89**, 529–537.

Ozgul, A., Childs, D.Z., Oli, M.K., Armitage, K.B., Blumstein, D.T., Olson, L.E., Tuljapurkar, S., & Coulson, T. (2010). Coupled dynamics of body mass and population growth in response to environmental change. *Nature*, **466**, 482–U5.

**
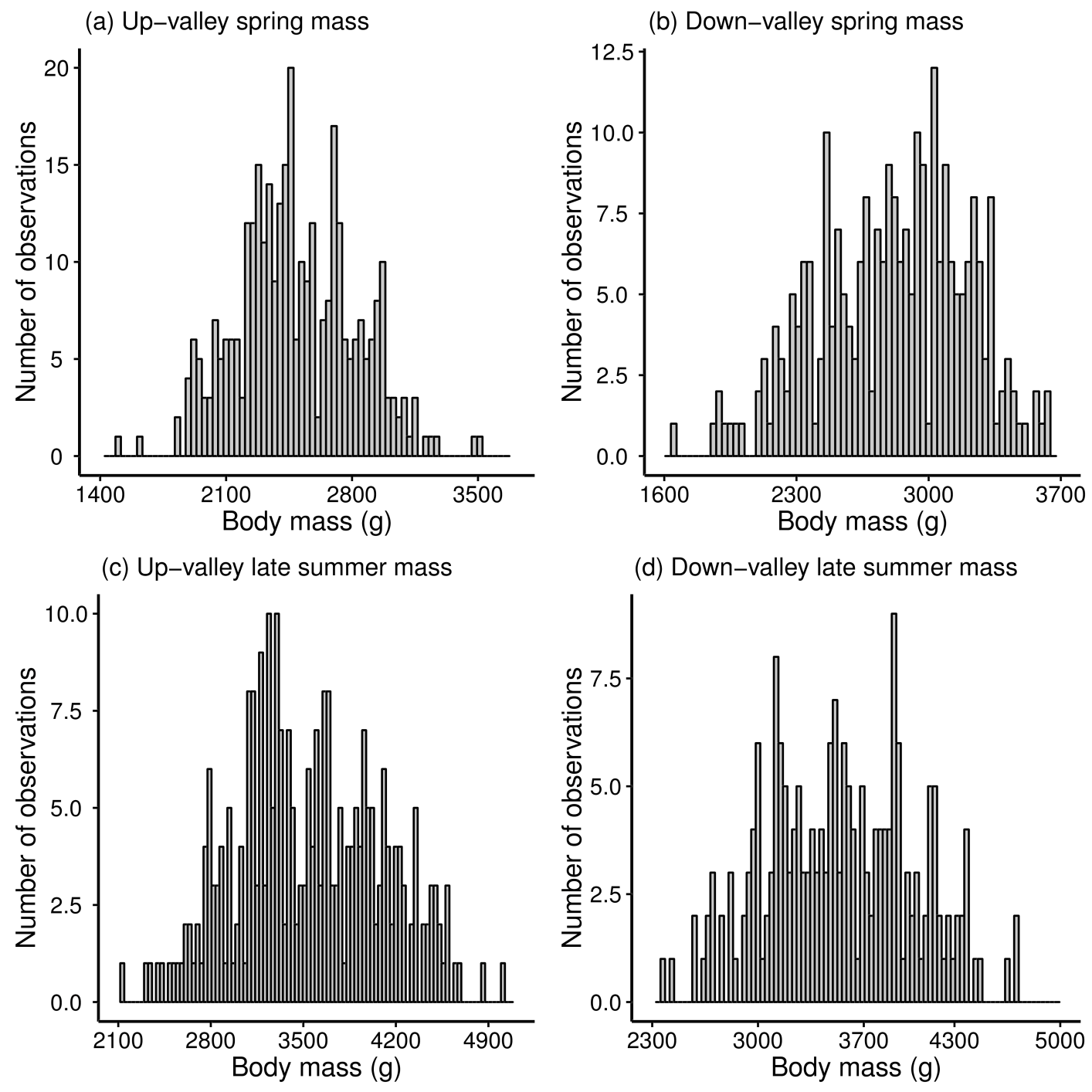
Figure S1**. Distribution of (a-b) spring and (c-d) late summer body mass of female yellow-bellied marmots living (a,c) up-valley and (b,d) down-valley. Lowest and highest (a) up-valley spring body masses = 1487g and 3514g respectively. Lowest and highest (b) down-valley spring body masses = 1637g and 3678g respectively. Lowest and highest (c) up-valley late summer body masses = 2126g and 4997g respectively. Lowest and highest (d) down-valley late summer body masses = 2374g and 4724g respectively.

**Table S1.** Spring and late summer body mass variation from age 9 and 8 respectively.

|  |  | | | | |  | | |
| --- | --- | --- | --- | --- | --- | --- | --- | --- |
|  | Spring | | | | | Late summer | | |
|  | *N* = 57; 28 individuals | | | | | *N* = 74; 36 individuals | | |
|  | |  | | |  | | | |
| Fixed effect | | Estimate (95% *CI*) | | | Estimate (95% *CI*) | | | |
|  | |  |  | |  | | |  |
| Intercept | | 3410.88 | | (3028.9/3773.8)* | | | 4298.03 | (3933.6/4659.1)* |
| Age | | -105.44 | | (-174.1/-31.0)* | | | -122.62 | (-200.8/-41.1)* |
| Valley[up] | | -860.91 | | (-1364.0/-347.8)* | | | -676.37 | (-1180.7/-165.3)* |
| Age x Valley[up] | | 115.69 | | (15.9/211.2)* | | | 94.40 | (-22.0/207.1) |

**Table S2.** Linear mixed-effects models quantifying effects of age, time to death (TTD), season, lifespan and reproduction (litter) on valley-specific body mass in female marmots. The reference levels for TTD, season (i.e. spring and late summer, LS) and litter are [0], [spring] and [no] respectively.

|  |  | | |  | |
| --- | --- | --- | --- | --- | --- |
|  | *N* = 457; 79 individuals | | | *N* = 608; 129 individuals | |
|  | Down-valley | | | Up-valley | |
|  |  | | |  | |
| Fixed effect | Estimate (95% *CI*) | | | Estimate (95% *CI*) | |
|  | | | |  | |
| Intercept | | 2733.50 | (2597.8 /2867.8)* | 2492.06 | (2383.7 /2600.2)* |
| Age | | 185.85 | (123.9 /248.0)* | 165.97 | (108.0 /223.8)* |
| Age² | | -10.74 | (-15.3 /-6.2)* | -8.35 | (-12.3 /-4.4)* |
| TTD[1] | | -5.93 | (-105.4 /93.7) | 33.32 | (-32.1 /98.5) |
| TTD[2] | | 99.79 | (-16.0 /215.6) | 17.73 | (-65.0 /100.2) |
| TTD[3+] | | 171.19 | (31.2 /311.1)* | 75.00 | (-46.5 /196.3) |
| Season[LS] | | 846.44 | (748.4 /944.5)* | 874.77 | (838.0 /911.6)* |
| Lifespan | | -6.72 | (-40.8 /27.2) | -19.28 | (-50.3 /11.7) |
| Litter[yes] | | -133.11 | (-197.5 /-68.4)* | -140.09 | (-188.3 /-91.5)* |
| TTD[1] x Season[LS] | | 235.20 | (98.2 /372.1)* |  |  |
| TTD[2] x Season[LS] | | 35.39 | (-109.0 /179.9) |  |  |
| TTD[3+] x Season[LS] | | 137.31 | (20.7 /253.9)* |  |  |
|  | |  |  |  |  |
|  | |  |  |  |  |
| Eliminated interactions | |  |  |  |  |
|  | |  |  |  |  |
| Age x Season[LS] | | -5.83 | (-25.4 /13.8) | -6.71 | (-24.0 /10.6) |
| Age² x Season[LS] | | -0.68 | (-2.2 /0.8) | -0.67 | (-2.0 /0.7) |
| TTD[1] x Season[LS] | |  |  | 29.85 | (-72.3 /132.0) |
| TTD[2] x Season[LS] | |  |  | -31.59 | (-140.6 /77.3) |
| TTD[3+] x Season[LS] | |  |  | 21.57 | (-71.3 /114.4) |
| Lifespan x Season[LS] | | -5.59 | (-24.2 /13.0) | -7.50 | (-20.8 /5.8) |
|  | |  |  |  |  |

**Table S3.** Down-valley and up-valley body mass variation from age 8 and 9 respectively (predicted ages at maximum body mass: down-valley = 8.0 years; up-valley = 8.6 years). The reference level for season (i.e. spring and late summer, LS) is [spring].

|  |  | | | | |  | | |
| --- | --- | --- | --- | --- | --- | --- | --- | --- |
|  | Down-valley | | | | | Up-valley | | |
|  | *N* = 86; 21 individuals | | | | | *N* = 45; 12 individuals | | |
|  | |  | | |  | | | |
| Fixed effect | | Estimate (95% *CI*) | | | Estimate (95% *CI*) | | | |
|  | |  |  | |  | | |  |
| Intercept | | 3192.72 | | (2897.8/3484.7)* | | | 2441.59 | (2054.9/2844.1)* |
| Age | | -81.37 | | (-140.5/-20.3)* | | | 32.66 | (-42.3/103.2) |
| Season[LS] | | 901.33 | | (783.1/1019.8)* | | | 1446.20 | (935.7/1962.1)* |
| Age x Season[LS] | |  | |  | | | -117.91 | (-220.6/-16.0)* |
|  | |  | |  | | |  |  |
| Eliminated interactions | |  | |  | | |  |  |
| Age x Season[LS] | | -86.24 | | (-176.8/3.6) | | |  |  |

**Table S4.** Eliminated interaction terms from spring and late summer body mass variation models.

|  |  | | |  | | | |
| --- | --- | --- | --- | --- | --- | --- | --- |
|  | Spring | | | Late summer | | | |
|  |  | | |  | | | |
| Fixed effect | Estimate (95% *CI*) | | | Estimate (95% *CI*) | | | |
|  |  |  | |  | | |  |
| Age x Valley[up] | 5.92 | | (-12.5 /24.4) | | 20.44 | (-6.6 /47.3) | |
| Age² x Valley[up] | 0.68 | | (-0.7 /2.0) | | 1.90 | (-0.2 /4.0) | |
| TTD[1] x Valley[up] |  | |  | | -122.72 | (-282.2 /36.9) | |
| TTD[2] x Valley[up] |  | |  | | -54.26 | (-227.7 /119.3) | |
| TTD[3+] x Valley[up] |  | |  | | -178.54 | (-336.4 /-20.1)* | |
| Age x TTD[1] | 0.22 | | (-15.6 /16.2) | | 12.66 | (-20.4 /45.8) | |
| Age x TTD[2] | 14.59 | | (-4.7 /34.1) | | 32.18 | (-8.6 /73.0) | |
| Age x TTD[3+] | 16.22 | | (-10.9 /43.9) | | 13.97 | (-35.1 /63.4) | |
| Age² x TTD[1] | 0.05 | | (-1.1 /1.2) | | 1.35 | (-1.0 /3.7) | |
| Age² x TTD[2] | 0.95 | | (-0.4 /2.3) | | 3.00 | (0.0 /6.0)* | |
| Age² x TTD[3+] | 1.00 | | (-1.0 /3.0) | | 1.90 | (-1.8 /5.6) | |
| Lifespan x Valley[up] | 6.58 | | (-19.1 /32.7) | | -23.26 | (-65.5 /19.0) | |

|  | | | Lifespan 4-6 | | | | | | Lifespan 7-9 | | | | | Lifespan 10-14 | | | | | |
| --- | --- | --- | --- | --- | --- | --- | --- | --- | --- | --- | --- | --- | --- | --- | --- | --- | --- | --- | --- |
|  | | | (*N* = 231; 95 individuals) | | | | | | (*N* = 190; 43 individuals) | | | | | | (*N* = 121; 17 individuals) | | | | |
|  |  | |  | | | | |  | | | | | | | |  | | | |
|  | Fixed effect | | Estimate (95% *CI*) | | | | | Estimate (95% *CI*) | | | | | | | | Estimate (95% *CI*) | | | |
|  |  |  | | |  | |  | | | |  | |  | | | | |  | |
|  | Intercept | 2777.25 | | | (2617.6 /2937.4)* | | 2661.28 | | | | (2454.1 /2865.5)* | | 2630.20 | | | | | (2435.4 /2825.0)* | |
|  | Age | 121.94 | | | (-68.9 /312.8) | | 226.59 | | | | (113.2 /340.2)* | | 137.09 | | | | | (87.9 /185.5)* | |
|  | Age² | 0.61 | | | (-20.1 /21.4) | | -17.39 | | | | (-28.6 /-6.2)* | | -5.85 | | | | | (-9.4 /-2.2)* | |
|  | TTD[1] | 63.69 | | | (-36.6 /163.3) | | 3.34 | | | | (-151.0 /159.2) | | 3.52 | | | | | (-116.6 /124.9) | |
|  | TTD[2] | 147.37 | | | (-2.3 /296.6) | | 75.60 | | | | (-123.1 /274.8) | | 190.22 | | | | | (60.0 /321.4)* | |
|  | TTD[3+] | 125.41 | | | (-90.2 /339.4) | | 92.10 | | | | (-126.2 /311.2) | | 220.37 | | | | | (88.3 /352.6)* | |
|  | Valley[up] | -238.00 | | | (-357.2 /-119.1)* | | -52.71 | | | | (-222.3 /121.8) | | -413.66 | | | | | (-658.9 /-174.7)* | |
|  | Litter[yes] | -31.09 | | | (-82.1 /19.6) | | -41.22 | | | | (-98.3 /15.2) | | -17.12 | | | | | (-75.9 /42.1) | |
|  | TTD[1] x Valley[up] | -18.24 | | | (-113.3 /76.9) | | -98.15 | | | | (-269.9 /72.1) | | 71.09 | | | | | (-126.6 /268.7) | |
|  | TTD[2] x Valley[up] | -114.13 | | | (-228.4 /0.1) | | -210.23 | | | | (-391.8 /-29.3)* | | -134.08 | | | | | (-323.9 /57.5) | |
|  | TTD[3+] x Valley[up] | -8.15 | | | (-152.4 /136.1) | | -228.94 | | | | (-380.3 /-78.5)* | | -90.73 | | | | | (-243.1 /65.3) | |
|  |  | | |  | |  | | | |  | |  | | | | |  | |  |

**Table S5.** Spring body mass variation models for females split into short- (4-6), medium- (7-9) and long-lived (10-14) lifespan categories.

|  | | Lifespan 4-6 | | | | | | Lifespan 7-9 | | | | | Lifespan 10-14 | | | | |
| --- | --- | --- | --- | --- | --- | --- | --- | --- | --- | --- | --- | --- | --- | --- | --- | --- | --- |
|  | | | (*N* = 171; 84 individuals) | | | | (*N* = 173; 40 individuals) | | | | | | | (*N* = 100; 16 individuals) | | | |
|  |  |  | | | | | |  | | | | |  | | | | |
|  | Fixed effect | Estimate (95% *CI*) | | | | | | Estimate (95% *CI*) | | | | | Estimate (95% *CI*) | | | | |
|  |  |  | | |  | |  | | |  | |  | | | |  | |
|  | Intercept | 3979.66 | | | (3701.8 /4259.7)* | | 3835.42 | | | (3519.2 /4150.0)* | | 3648.86 | | | | (3336.1 /3959.9)* | |
|  | Age | 112.74 | | | (-337.3 /557.2) | | 212.96 | | | (22.6 /401.8)* | | 142.25 | | | | (26.9 /258.3)* | |
|  | Age² | 7.74 | | | (-42.4 /58.6) | | -11.41 | | | (-30.1 /7.3) | | -6.44 | | | | (-15.1 /2.2) | |
|  | TTD[1] | 162.97 | | | (1.9 /322.9)* | | -2.26 | | | (-194.0 /189.7) | | 267.23 | | | | (39.0 /499.0)* | |
|  | TTD[2] | 106.46 | | | (-138.8 /354.8) | | -96.16 | | | (-353.7 /161.2) | | 311.52 | | | | (59.4 /571.4)* | |
|  | TTD[3+] | 293.90 | | | (-60.5 /655.7) | | 76.28 | | | (-250.3 /401.8) | | 401.52 | | | | (120.8 /684.7)* | |
|  | Valley[up] | -384.15 | | | (-571.4 /-197.4)* | | -274.89 | | | (-495.7 /-46.8)* | | -626.04 | | | | (-847.4 /-408.2)* | |
|  | Litter[yes] | -296.69 | | | (-402.6 /-190.9)* | | -324.29 | | | (-418.5 /-229.9)* | | -296.41 | | | | (-417.5 /-171.5)* | |
|  |  | | |  | |  | | |  | |  | | | |  | |  |

**Table S6**. Late summer body mass variation models for females split into short- (4-6), medium- (7-9) and long-lived (10-14) lifespan categories.
